# A novel recessive *PDZD7* bi-allelic mutation in an Iranian family with non-syndromic hearing loss

**DOI:** 10.1186/s12920-021-00884-4

**Published:** 2021-02-02

**Authors:** Hossein Fahimi, Samira Behroozi, Sadaf Noavar, Farshid Parvini

**Affiliations:** 1 Department of Genetics, Faculty of Advanced Science and Technology, Tehran Medical Sciences, Islamic Azad University, Tehran, Iran; 2 Pharmaceutical Sciences Research Center, Tehran Medical Sciences, Islamic Azad University, Tehran, Iran; 3 Department of Biology, Faculty of Basic Sciences, Semnan University, Semnan, 35131-19111 Iran

**Keywords:** ARNSHL, Targeted exome sequencing, *PDZD7* gene, Missense mutation, Iran

## Abstract

**Background:**

Autosomal recessive non-syndromic hearing loss (ARNSHL) is genetically and phenotypically heterogeneous with over 110 genes causally implicated in syndromic and non-syndromic hearing loss. Here, we investigate the genetic etiology of deafness in two *GJB2* and *GJB6* negative patients presenting with pre-lingual, progressive, severe hearing loss.

**Methods:**

Targeted exome sequencing (TES) using Next Generation Illumina Sequencing was used to analyze the exonic and some other important genomic regions of 154 genes in the proband. Subsequently, the mutation found was confirmed by Sanger sequencing in other affected sibling and healthy family members. The possible impact of the reported mutation on the corresponding protein was also evaluated by using bioinformatics tools. Moreover, the affected patients underwent audiological and ophthalmic evaluations.

**Results:**

TES identified a novel homozygous missense mutation c.251T>C (p.I84T) in exon 3 of *PDZD7* gene. In addition, segregation and phenotype-genotype correlation analysis as well as *in-silico* evaluations confirmed the autosomal recessive inheritance pattern and disease-causing nature of mutation found.

**Conclusions:**

In overall, our finding could expand the pathogenic mutations spectrum and strengthens the clinical importance of the *PDZD7* gene in ARNSHL patients. It can also aid to conduct genetic counseling, prenatal diagnosis and clinical management of these types of genetic disorders.

## Background

Hearing loss (HL) is a most common sensori-neural disorder which is clinically and genetically heterogeneous. This defect is categorized in two types, syndromic (30%) and non-syndromic (70%) [1]. Mutations in more than 110 genes have been listed which can cause syndromic or non-syndromic HL (https://hereditaryhearingloss.org/). Mutations of *GJB2* and *GJB6* genes have been reported as the most causative subjects. Nonetheless, patients who are negative for mutation in *GJB2*/*GJB6* genes are considered as attractive subjects to investigate the genetic basis of disease. One of the recently identified genes is PDZ domain-containing 7 (*PDZD7*) gene (MIM *612971). The *PDZD7* encodes a PDZ domain containing scaffold protein that highly expressed in hair cells of inner ear. This protein is also expressed in the cilia of photoreceptors [2, 3]. PDZ domain of PDZD7 protein is responsible for binding with other proteins such as VLGR1, WHRN, and USH2A which all are very important in development and proper function of auditory and visual systems [4]. *PDZD7* is highly homologous with two proteins, harmonin and whirlin, with similar expression patterns. These two proteins are also scaffolding proteins that give rise to autosomal recessive non-syndromic hearing loss (ARNSHL) and Usher syndrome(USH) disorders when mutated [5–7]. Defects in these proteins can damage hearing and vision. The relevant disorders are described as different types of Usher syndrome, non-syndromic hearing loss, and non-syndromic retinitis pigmentosa (RP) [5, 8–12]. It has been shown that PDZD7 play a vital role in hearing by normal development, morphology, and function of stereo-cilia of mice ear cells [4, 7, 13]. It has been reported that dysfunction or mutations of *PDZD7* results in Usher syndrome type 2 (USH2) [5] and ARNSHL [12, 14–16].

In short, the *PDZD7* gene comprised of 16 exons located on chromosome 10 (10q24.31) which encodes PDZD7 with 1033 amino acid. PDZD7 folds in to four domains (three PDZ and one Harmonin domain). PDZ2 domains are involved in dimerization of PDZD7 and also interact with VLGR1 and WHRN [4]. Herein, we report a novel homozygous missense mutation of *PDZD7* in two Iranian patients with ARNSHL and negative for *GJB2* and *GJB6* genes mutation. This finding can expand the genetic spectrum and detection methods of the corresponding abnormalities and help physicians to order more inclusive tests. Furthermore, such studies shows that advances in the field of next-generation sequencing allow for a more accurate and less expensive diagnosis and study of molecular basis of hearing loss [17, 18].

## Methods

At the present research we explored the molecular mechanism of ARNSHL in two affected patients from a single family. This study was approved by the Committee of Ethics in Biomedical Researches of Islamic Azad University of Medical Sciences, IR.IAU.PS.REC.1396.91. Written informed consent was obtained from all patients and their family members who participated in this study.

### Patients

**Fig. 1:**
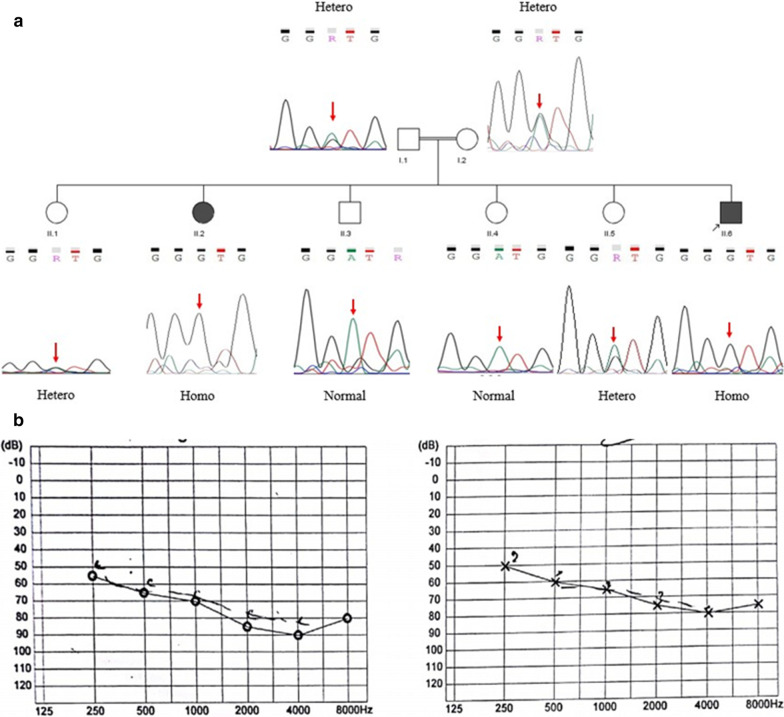
Family pedigree and audiologic output of the proband (II-6). **a** Pedigree and segregation analysis of the *PDZD7* gene mutation c.251T>C: p.I84T in a family with ARNSHL. Both patients (II-2 and II-6) are homozygote mutants and their parents are heterozygote. Sanger sequencing of four normal siblings of the patients revealed that individuals II-3 and II-4 are homozygous wild type whereas other two siblings (II-1 and II-5) are heterozygote. **b** Audiograms of the affected proband. The left chart is for right ear and the chart in right is for left ear. Audiograms were obtained using pure tone audiometry with air conduction from frequencies 250 to 8000 Hz

The studied family is of Iranian origin located in Semnan province. The two affected individuals are included in this family with two common ancestors (Fig. 1). The proband was a 30-year-old man with autosomal ARNSHL without any vision damage. He is a product of consanguineous marriage and has a sister (23-year-old) with the same phenotype. The subjects underwent audiological and ophthalmic evaluations.

### Screening of *GJB2* and *GJB6* genes

Blood samples were collected and genomic DNAs of the all participants were extracted from peripheral blood samples using QIAamp DNA Blood Mini Kit Cat No. 51104 (QIAGENE, Germany) according to the manufacturer’s instructions. First of all, the patients were screened for most common mutation c.35delG of *GJB2* gene using allele specific PCR (PCR master mix, Cat no. 180301 (Ampliqon, Denmark)). Since the patients were negative for the 35delG mutant allele, the entire non-coding (exon 1), coding (exon 2) and flanking intronic regions of the *GJB2* gene were amplified and directly sequenced using following primers: CXF’ 5′ CCCTCCGTAACTTTCCCAGT 3′/CXR’ 5′ AAAACGTTTGGTGGCAGTGG 3′ (607 bp) for amplification of exon 1 and CXF 5′ CTCCCTGTTCTGTCCTAGCT 3′/CXR 5′ CTCATCCCTCTCATGCTGTC 3′ (809 bp) for amplification of exon 2. The PCR reactions were subjected to an initial denaturation step 95 °C/10 s, followed by 40 cycles of 95 °C/30 s, 60 °C/1 min and 72 °C/30 s. Then, based on negative results of Sanger sequencing for *GJB2* gene, the patients were screened for two known deletions del(D13S1830) and del(D13S1854) of *GJB6* gene.

### Targeted exome sequencing and bioinformatics analysis

Regarding to negative result for screening of *GJB2* and *GJB6* genes mutations, the patient II-6 (proband) was more investigated by targeted exome sequencing (TES) to enrich all exons of 154 protein-coding genes (the annotated genes causing hearing impairment in the OMIM database, please see the Additional file 1) as well as some other important genomic regions (exon–intron boundaries such as splice regions) involved in hearing. The TES performed using Agilent V.6 kit (Agilent, USA) on an Illumina platform using Illumina NextSeq500 instrument. The sequencing depth was 100X. Filtering at the first step was based on frequency. Then, intronic, upstream, downstream, 3′-UTR, 5′-UTR, intergenic and other non-coding variants were filtered. After that, synonymous mutations were also filtered. Generally, test platform examined more than 95% of the targeted regions with sensitivity of > 99%. By TES test, point mutations, micro-insertions/deletions and micro-duplications (< 20 bp) can be simultaneously detected. The obtained results were analyzed by open access software, namely GATK [19], BWA aligner [20], and annovar [21]as well as public databases gnomAD, ExAC, GME and Kaviar (~ Known VARiants). Furthermore, ACMG guidelines and local population database with more than 3000 unrelated individuals (BayanGene) were utilized. Ninety healthy individuals with the same ethnicity as the studied patients were also screened for the mutation found. We used Provean, SIFT, Mutation Assessor, CADD-phred, and Polyphen software to predict the possible pathogenicity of the mutation found. To analyze the evolutionary conservation of amino acid changed, the mutated PDZD7 sequence was aligned with the GenBank reference sequence (NM_001195263) and its orthologs from *Macaca mulatta*, *Canis lupus, Bos taurus, Mus musculus, Rattus norvegicus, Gallus gallus, Danio rerio, and Xenopus tropicalis* species using Clustal Omega online software. Furthermore, structural 3D models for PDZ1 domain in the PDZD7 protein were constructed based on homology modeling and by using SWISS-MODEL server (https://swissmodel.expasy.org) and were viewed using PyMOL program.

### Sanger sequencing and segregation analysis

To confirm the novel mutation found in the *PDZD7* gene, PCR and Sanger sequencing were performed for both patients and their normal family members using following primers: F-5′ TCCCTGACAGCAGCATCC 3′ and R- 5′ GCCTTAGAAATGGGCTGACCTG 3′ (PCR product: 385 bp). Finally, the sequencing data was analyzed using Chromas software.

## Results

**Fig. 2:**
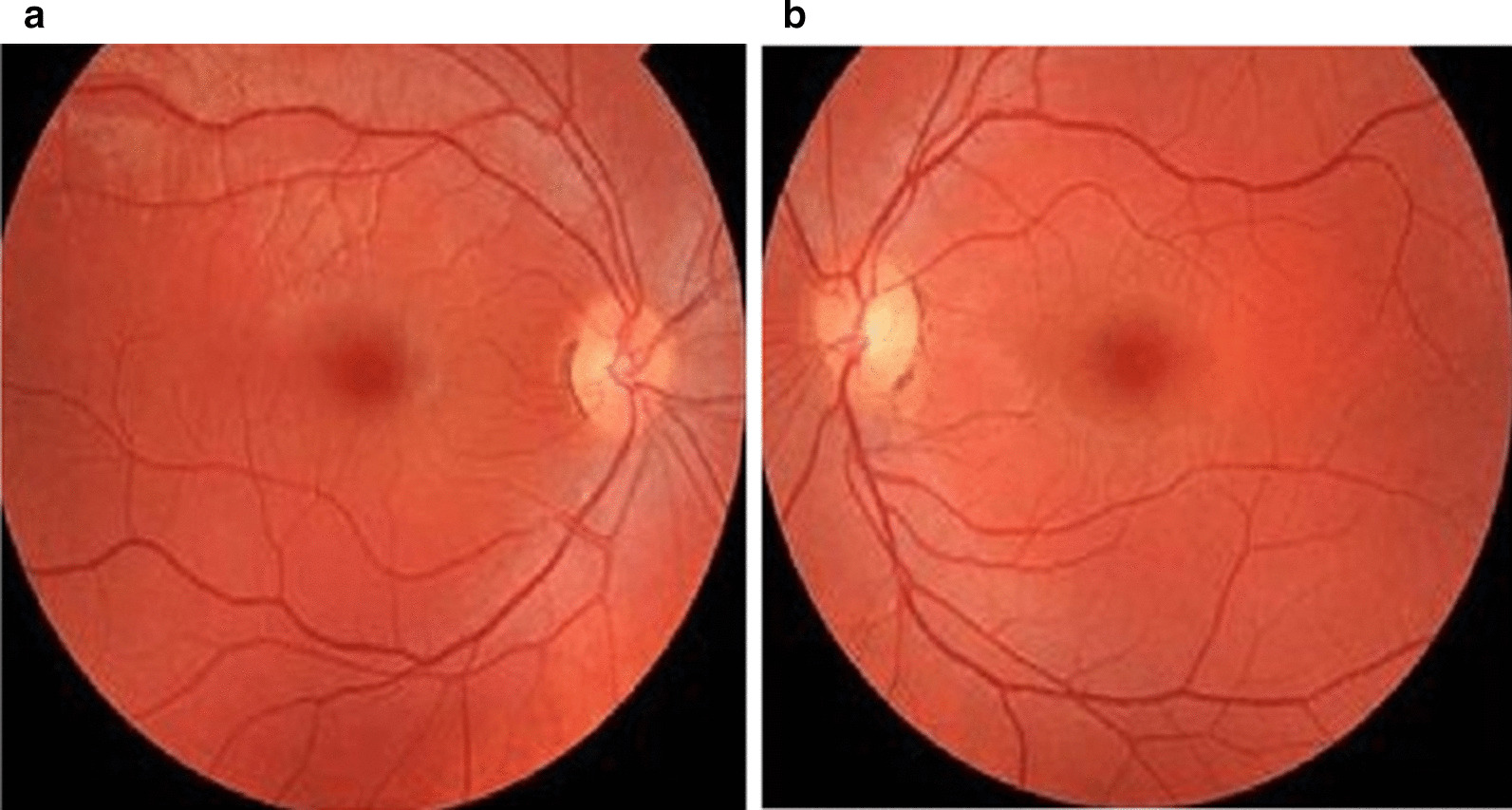
Image of the normal fundus of the proband ‘s right (**a**) and left (**b**) eyes

Regarding to proband (patient II-6) who was negative for *GJB2* and *GJB6* genes mutations, he was investigated by TES technique. A novel homozygous missense mutation was identified in exon3 of the *PDZD7* gene (NM_001195263); chr10:101024044A/G: c.251T>C: p.I84T. Our review of public databases and the local population database didn’t identify any previous reports of the mutation found. Additionally, none of 90 screened healthy controls showed this mutation confirming the novelty of the mutation found. This point mutation results in replacement of a threonine instead of isoleucine 84 in PDZD7 protein. Furthermore, the Sanger sequencing confirmed the mutation found and co-segregated with autosomal recessive inheritance pattern of NSHL disorder (Fig. 1). As expected, affected sister (II-2) had the identified mutation in the *PDZD7* gene and unaffected parents (I.1 and I.2 in pedigree) of the proband were heterozygous carriers. Additionally, segregation analysis showed that two normal siblings (II-3 and II-4) of the patients were homozygous wild type whereas other two normal siblings (II-1 and II-5) were heterozygous for the missense mutation c.251T>C: p.I84T of *PDZD7* gene. The corresponding family pedigree and the results of Sanger sequencing are illustrated in Fig. 1. With respect to audiological and ophthalmic evaluations, while the ophthalmic exams of two studied HL patients were normal (Fig. 2), their pure tone audiometry at 250–8000 Hz, showed severe down sloping hearing loss (Fig. 1).

**Fig. 3:**
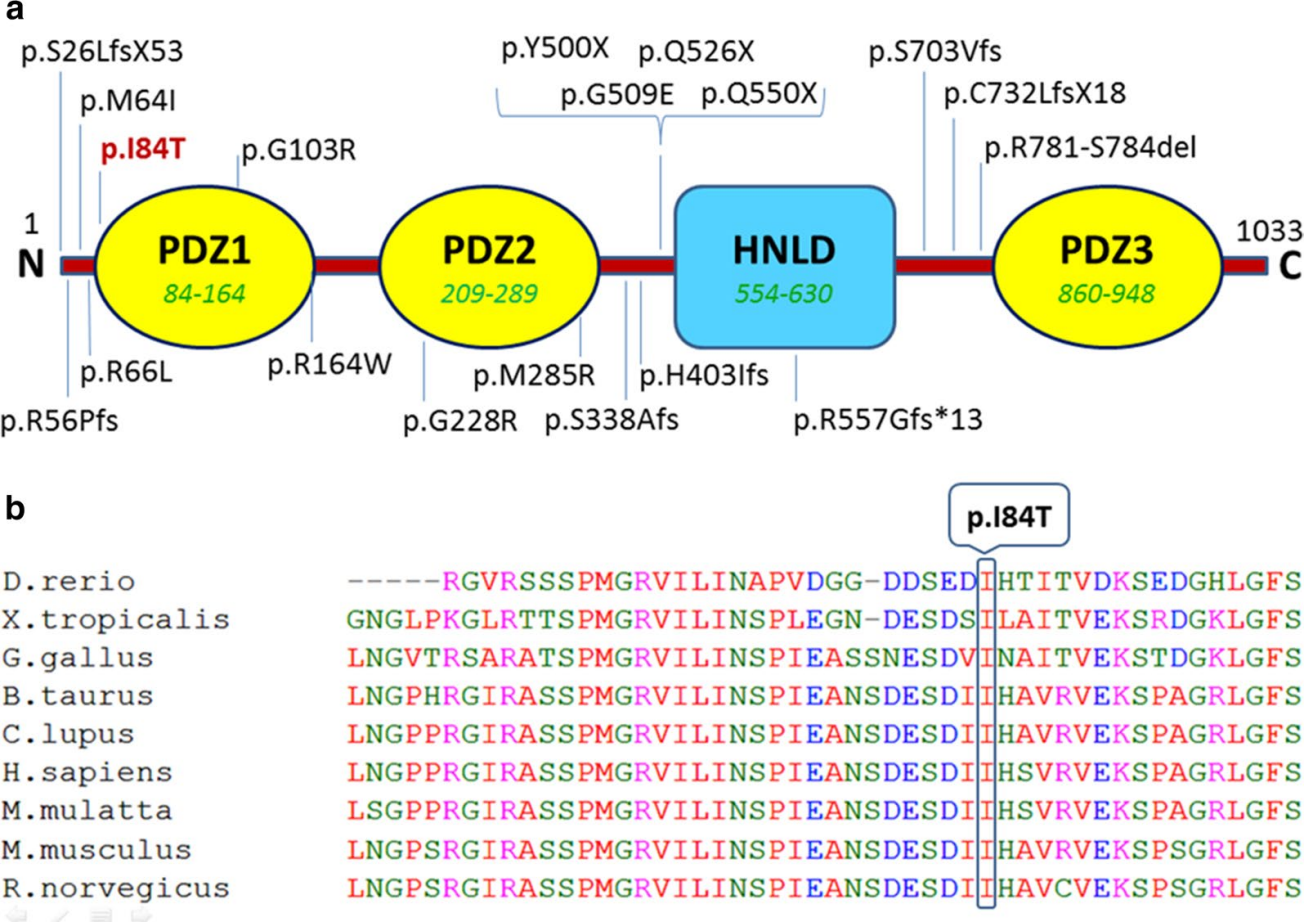
A schematic representation of the PDZD7 protein domains and the identified mutation p.I84T. **a** PDZD7 comprised of three conserved PDZ domains (yellow) and one HNLD domain (blue). All previously reported PDZD7mutations are shown and the new reported mutation is depicted in red. **b** Multiple alignment of PDZD7 protein sequence shows that the Ile residue at situation 84 is highly conserved across nine species

Furthermore, we performed *in-silico* analyses for the identified missense mutation, and it was predicted to be “Deleterious” by PROVEAN, SIFT, Polyphen, and Mutation Taster (Table 1). These data revealed that this novel mutation could be cause of deafness in this family. As depicted in Fig. 3a, PDZD7 comprised of four conserved domains (three PDZ domains and one harmonin-N-like domain (HNLD)) [4]. The results of multiple alignment for the corresponding sequence revealed that the c.251T>C (p.I84T) occurs at highly conserved residues in PDZ1 (Fig. 3b).

**Table 1:** Prediction of pathogenicity of the homozygous mutation c.251T>C in the *PDZD7* gene

Gene (RefSeq)	Mutation	PROVEAN	SIFT	Polyphen	Mutation Taster	Mutation Assessor	CAAD-Phred	Phenotype
PDZD7 (NM_001195263)	c.251T>C (I84T)	D	D	D	D	L	19.25	ARNSHL

D, damaging; L, low pathogenicity; ARNSHL, autosomal recessive non-syndromic hearing loss

**Fig. 4:**
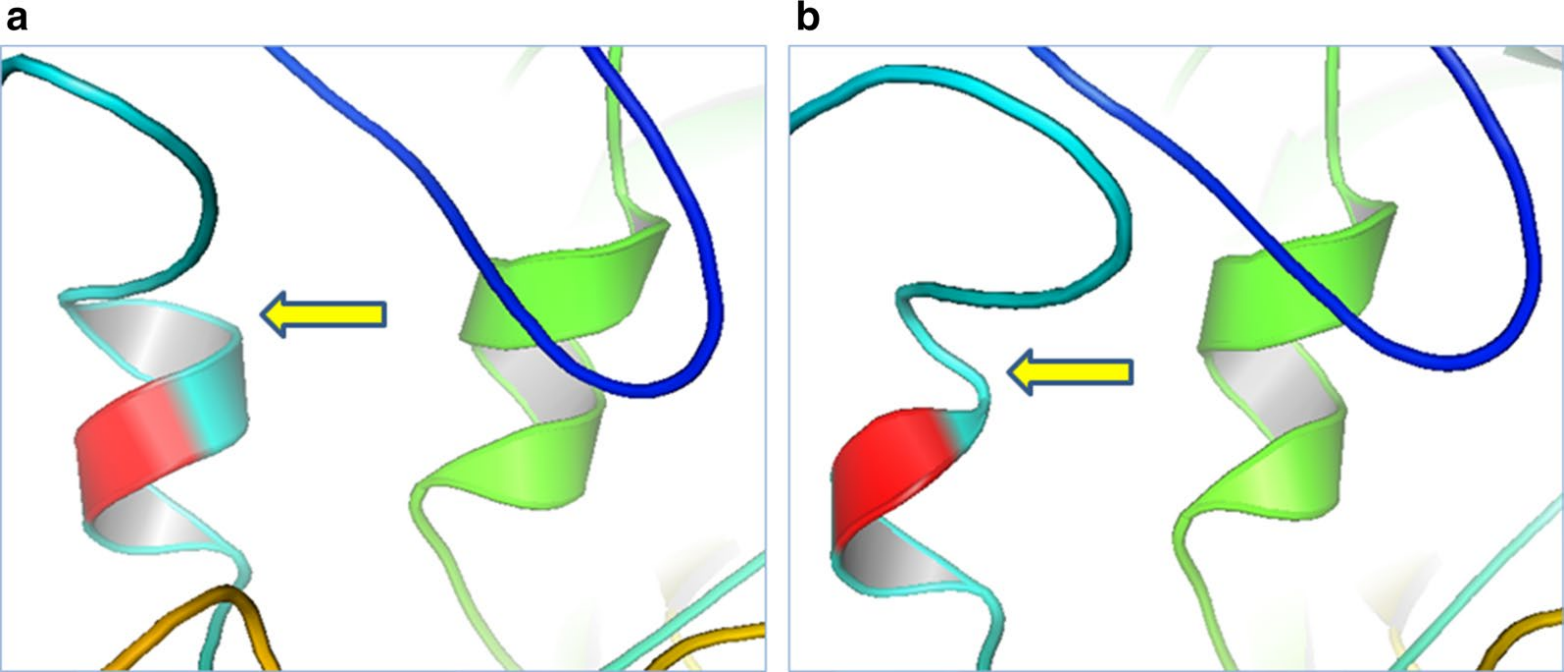
A schematic representation of PDZD7 and structural analysis of the normal and mutant variants. The 3D structural models were constructed by SWISS-MoDEL and viewed with PyMol software. As it is locally zoomed (at 12 Angstrom) the position of mutation (residue 84 that highlighted in red) makes a complete round of alpha-helix structure in normal protein (**a)**. As shown by a yellow arrow, substitution of isoleucine to threonine at position 84 resulted in formation of a truncated alpha-helix in mutant PDZD7 protein (**b**)

In order to determine how the novel p.I84T mutation affects the conformation of PDZD7, 3D structural models of both wild type and mutant PDZD7 were constructed by SWISS-MODEL. The sequence identity between the target and template was 23.79%. The predicted models covered the target sequence of PDZD7 (1–293) and were viewed with PDB viewer software (PyMol). Since isoleucine is a non-polar amino acid whereas threonine is a polar amino acid, it was predicted that substitution of isoleucine to threonine at position 84, possibly perturbing the non-covalent interactions between residues and resulted in formation of a truncated alpha-helix in mutant PDZD7 protein (Fig. 4).

## Discussion

The *PDZD7* gene is highly conserved in human, Rhesus monkey, dog, cow, rat, chicken, zebrafish, and frog (https://www.ncbi.nlm.nih.gov/homologene/129509). This gene comprised of 17 exons which encodes a large scaffold protein named PDZD7. As previously mentioned, the *PDZD7* is expressed in retinal photoreceptors and inner ear hair cells [4, 13]. Along with other proteins such as WHRN, USH2A, and GPR98, PDZD7 play a critical role in formation of USH2 quaternary complex which is important in normal development and proper function of both auditory and visual systems [4]. Abnormal expression of the *Pdzd7* gene results in improper organization of hair bundles in mice which can lead to hearing loss [13].

To date, 22 different mutations of the *PDZD7* gene have been reported in patients with ARNSHR from Iran, Pakistan, Germany, Korea, Canada, and China. The type of these mutations and the resulted phenotypes are summarized in Table 2. Of course, the *PDZD7* gene was originally reported as being responsible for non-syndromic congenital sensori-neural hearing loss in a 9-year-old German boy due to a reciprocal translocation t(10;11)(q24.3;q23.3) [16]. Affected siblings of the present study shows bilaterally symmetrical severe down sloping ARNSHL. The next generation sequencing using TES was identified a novel homozygous mutation c.251T>C: p.I84T of *PDZD7* gene. Furthermore, segregation analysis determined that this substitution of threonine to Isoleucine at highly conserved position 84 is causative (Fig. 1) and affects the function of protein deleteriously. This mutation is located in the PDZ1 domain (Fig. 3). It has been previously documented that PDZ1 domain plays a critical role in interaction of PDZD7 with cytoplasmic part of USH2A protein [4]. Therefore, the novel p.I84T mutation may interrupt normal interaction between PDZD7 and USH2A. As recently reported, PDZD7-binding proteins, such as ADGRV1, gelsolin, and β-catenin, play significant roles in hearing. PDZD7 interacts with these proteins via domains PDZ1/PDZ2 [22]. On the other hands, *in-silico* analysis of normal and mutant PDZD7 protein models revealed that isoleucine substitution with threonine may results in formation of a truncated alpha-helix in mutant PDZD7 (Fig. 4). Although in silico analysis of the mutant protein is a reliable prediction tool for structural impacts of missense mutations, more experimental evaluations are actually needed to completely determine the consequences of the detected mutation on expression and function of the corresponding gene and protein.

**Table 2:** Summary of the all reported *PDZD7* mutations and associated phenotypes to date

Nucleotide change	Consequence	Genotype	The affected Domain	Phenotype	Family origin	References
c.166_167insC	p.R56PfsX24	Hetero	–	USH2	Canadian	[5]
c.1750–2A>G	Splice site	Hetero	HNL		German	
c.2194_2203del	p.C732LfsX18	Hetero	–			
c.76_77del	p.S26LfsX53	Hetero	–	HL	Korean	[23]
c.307G>C	p.G103R	Homo	PDZ1	ARNSHL	Iranian	[12]
c.682G>A	p.G228R	Homo	PDZ2			
c.1576C>T	p.Q526X	Homo	–			
c.854T>G c.1500C>A	p.M285R p.Y500X	Comp Het	PDZ2 –			
c.1648C>T c.2107delA	p.Q550X p.S703Vfs	Comp Het	–	ARNSHL	German	[15]
c.226+2_226+ 5delTAGG	Abolished splicing in exon 2—intron 2 junction	Homo	–	ARNSHL	Pakistani	[24]
c.197G>T	p.R66L	Homo	– -	ARNSHL	Chinese	[14]
c.1207delC c.166-167insC^a^	p.H403Ifs p.R56Pfs	Comp Het				
c.1012delA	p.S338Afs	–	–	Usher syndrome, type 2A	–	ClinVar ID: 560723
c.192G>A c.1648C>T c.2341_2352del	p.M64I p.Q550X p.R781-S784del	Comp Het	–	ARNSHL	Chinese	[25]
c.490C>T c.1669delC c.1526G>A	p.R164W p.R557Gfs*13 p.G509E	Homo/Comp Het	PDZ1 and HNLD	ARNSHL	Korean	[26]
c.251T>C	p.I84T	Homo	PDZ1	ARNSHL	Iranian	This report

The first reported mutation in *PDZD7* was detected by Schneider et al. as a consequence of a homozygous reciprocal translocation, t(10;11) [16]

^a^This frame-shift mutation was originally reported by Eberman et al. (5)

Although, the gene encoding PDZD7 protein was originally defined as a modifier of retinal disease and a contributor for Usher syndrome [5, 16], but this interpretation was revised according to the recent studies [12, 14]. In accordance with this recent study, mutation in PDZD7 may results in NSHL without any RP manifestations. It could be because of compensatory effects of other scaffolding proteins involved in retina function [12].

## Conclusions

Overally, this report supports the contribution of *PDZD7* bi-allelic mutations to the etiology of ARNSHL in mankind and extends the mutations spectrum of the *PDZD7* gene in Iranian population and also worldwide. Additionally, this research is considerable because it leads to a better understanding of *PDZD7* recessive mutations on phenotypic outcome and strengthens the clinical importance of this gene in ARNSHL patients.

## Supplementary Information

**Additional file 1.** The list of analyzed genes involved in Hearing impairment.

## Data Availability

The datasets generated and/or analysed during the current study have uploaded in the NCBI dbVar repository (https://www.ncbi.nlm.nih.gov/clinvar/variation/992657/). The direct web links to the GenBank reference sequence NM_001195263 (https://www.ncbi.nlm.nih.gov/nuccore/NM_001195263.1) and its orthologs from Macaca mulatta (https://www.ncbi.nlm.nih.gov/nucleotide/XM_015148033.2?report=genbank&log$=nucltop&blast_rank=12&RID=Z9R3J22J013), Canis lupus (https://www.ncbi.nlm.nih.gov/nucleotide/XM_035707965.1?report=genbank&log$=nucltop&blast_rank=65&RID=Z9R3J22J013), Bos taurus (https://www.ncbi.nlm.nih.gov/nuccore/XM_005225682.4), Mus musculus (https://www.ncbi.nlm.nih.gov/nuccore/NM_001195265.1), Rattus norvegicus (https://www.ncbi.nlm.nih.gov/nuccore/XM_006231449.3), Gallus gallus (https://www.ncbi.nlm.nih.gov/nuccore/XM_015288750.2), Danio rerio (https://www.ncbi.nlm.nih.gov/nuccore/NM_001190757.1), and Xenopus tropicalis (https://www.ncbi.nlm.nih.gov/nuccore/XM_031906428.1) species are all available.

## Abbreviations

ARNSHL: autosomal recessive non-syndromic hearing loss
HL: hearing loss
*GJB2*: gap junction beta-2 protein
*GJB6*: gap junction beta 6 protein
TES: targeted exome sequencing
*PDZD7*: PDZ domain-containing 7
USH: Usher syndrome
RP: retinitis pigmentosa
USH2: Usher syndrome type 2
HNLD: harmonin-N-like domain
