# Supplementary material for: A novel recessive PDZD7 bi-allelic mutation in an Iranian family with non-syndromic hearing loss
Source: BMC Med Genomics. 2021 Feb 2;14:37. doi: 10.1186/s12920-021-00884-4 (PMC7852090; doi:10.1186/s12920-021-00884-4)
Supplement: Supplementary file 1 — Additional file 1. The list of analyzed genes involved in Hearing impairment. [file 12920_2021_884_MOESM1_ESM.pdf]

ABHD12				
ACTG1	EDNRB	MARVELD2	OTOA	TIMM8A
ALMS1	ESPN	MIR96	OTOF	TJP2
ATP2B2	ESRRB	MIRN96	OTOG	TMC1
BSND	EYA1	MITF	OTOGL	TMIE
CACNA1D	EYA4	MSRB3	PAX2	TMPRSS3
CCDC50	FGF3	MT_CO1	PAX3	TNFRSF11B
CCOL11A1	FGFR1	MT_CO2	PCDH15	TPRN
CDH23	FGFR2	MT_CYB	PDSS1	TRIOBP
CEACAM16	FGFR3	MT_ND1	PDZD7	USH1C
CHD7	FOXI1	MT_ND4	PHEX	USH1G
CIB2	GATA3	MT_ND6	POU3F4	USH2A
CLDN14	GFER	MT_RNR1	POU4F3	WFS1
CLRN1	GIPC3	MT_TC	PRPS1	
COCH	GJA1	MT_TE	PRRX1	
COL11A1	GJB2	MT_TF	PTPRQ	
COL11A2	GJB3	MT_TH	RDX	
COL2A1	GJB6	MT_TK	SEMA3E	
COL4A3	GLI3	MT_TL1	SERAC1	
COL4A4	GPR98	MT_TS1	SERPINB6	
COL4A5	GPSM2	MT_TS2	SIX1	
COL9A1	GRHL2	MT_TV	SIX5	
COL9A2	GRXCR1	MT_TW	SLC17A8	
CRYM	HGF	MTATP8	SLC19A2	
DFNA5	HOXA1	MTCO1	SLC26A4	
DFNB31	HOXA2	MTND1	SLC26A5	
DFNB59	IGF1	MTRNR1	SLC4A11	
DFNB59/PJVK	ILDR1	MYH14	SMAD4	
DIABLO	KCNE1	MYH9	SMPX	
DIAPH1	KCNJ10	MYO15A	SNAI2	
DIAPH3	KCNQ1	MYO1A	SOBP	
DLX5	KCNQ4	MYO3A	SOX10	
DSPP	LHFPL5	MYO6	SOX9	
E160	LOXHD1	MYO7A	STRC	
E160:E161	LRP2	NDP	TCOF1	
E161	LRTOMT	OPA1	TECTA	
EDN3				
